# Molecular Dynamics of Lipopolysaccharide-Induced Lung Injury in Rodents

**DOI:** 10.3389/fphys.2020.00036

**Published:** 2020-02-05

**Authors:** Hannes Domscheit, Maria A. Hegeman, Niedja Carvalho, Peter M. Spieth

**Affiliations:** ^1^Department of Anesthesiology and Critical Care Medicine, University Hospital Dresden, Technische Universität Dresden, Dresden, Germany; ^2^Laboratory of Experimental Intensive Care and Anesthesiology (L∙E∙I∙C∙A), Department of Intensive Care, Academic Medical Center, Amsterdam, Netherlands; ^3^Department of Educational Consultancy and Professional Development, Faculty of Social and Behavioral Sciences, Utrecht University, Utrecht, Netherlands

**Keywords:** acute respiratory distress syndrome, inflammation, lipopolysaccharide-induced lung injury, dynamics, toll-like receptor 4, time-dependent

## Abstract

Acute respiratory distress syndrome (ARDS) is a common disease entity in critical care medicine and is still associated with a high mortality. Because of the heterogeneous character of ARDS, animal models are an insturment to study pathology in relatively standardized conditions. Rodent models can bridge the gap from *in vitro* investigations to large animal and clinical trials by facilitating large sample sizes under physiological conditions at comparatively low costs. One of the most commonly used rodent models of acute lung inflammation and ARDS is administration of lipopolysaccharide (LPS), either into the airways (direct, pulmonary insult) or systemically (indirect, extra-pulmonary insult). This narrative review discusses the dynamics of important pathophysiological pathways contributing to the physiological response to LPS-induced injury. Pathophysiological pathways of LPS-induced lung injury are not only influenced by the type of the primary insult (e.g., pulmonary or extra-pulmonary) and presence of additional stimuli (e.g., mechanical ventilation), but also by time. As such, findings in animal models of LPS-induced lung injury may depend on the time point at which samples are obtained and physiological data are captured. This review summarizes the current evidence and highlights uncertainties on the molecular dynamics of LPS-induced lung injury in rodent models, encouraging researchers to take accurate timing of LPS-induced injury into account when designing experimental trials.

## Introduction

Acute respiratory distress syndrome (ARDS) is a common but often under-recognized and under-treated disease in critical care medicine and is still associated with high morbidity and mortality (Bellani et al., 2016). ARDS can be caused either by direct lung injury (e.g., pneumonia, aspiration) or extra-pulmonary diseases, affecting the lung secondarily (e.g., sepsis, pancreatitis). The severity of ARDS may differ among patients and may change during the course of the disease (Ranieri et al., 2012). Up to every tenth patient admitted to the intensive care unit presents with respiratory failure consistent with ARDS (Rubenfeld et al., 2005). The heterogeneous pathophysiology of ARDS makes it difficult to identify pathophysiological mechanisms and specific therapeutic interventions. By standardizing observational conditions, animal models facilitate mechanistic studies and provide insights into the pathophysiology of ARDS. The choice of animal species depends on the pathomechanism to be studied. Rodent models of LPS injury have been widely used due to easy availability, housing, and relatively low costs (Redl et al., 1993; Lewis et al., 2016). In addition, the monitoring of hemodynamic and pulmonary mechanics parameters has become easier using special miniaturized equipment. Disadvantages of using small animals are, e.g., the small volume of blood and the reduced sensitivity to endotoxemia when compared to other species such as pig and sheep (Kohman et al., 2010). The use of sheep is prominent in studies involving microvascular pathophysiology and pulmonary permeability due to easy access to the pulmonary lymphatic system and increased lymphatic flow in response to small doses of endotoxin (Wiener-Kronish et al., 1991; Redl et al., 1993). Despite the pigs’ similarities to humans in relation to the anatomy, genetics, and physiology, the LPS challenge is usually performed as indirect lung injury by intravenous, intraperitoneal, or intramuscular administration (Wyns et al., 2015). Furthermore, the existence of the fibroproliferative phase of ALI/ARDS in pig LPS models is unclear (Wang et al., 2008).

The purpose of animal models is to mimic human disease. An ideal animal model of ARDS should reproduce all pathophysiological features of human ARDS (Matute-Bello et al., 2011). However, not all complex features of ARDS and coexisting diseases can be reproduced in animal models (Matute-Bello et al., 2008). Most ARDS models reproduce either the acute inflammatory phase or chronic fibroproliferative phase depending on the specific research question (Matute-Bello et al., 2011). So, to be able to draw clinically meaningful conclusions, individual dynamics of the model must be taken into account. The similarities and differences between existing animal models and clinical ARDS have been reported previously (Matute-Bello et al., 2011). Moreover, technical issues with individual models were described (Matute-Bello et al., 2011). The most practical and invariable small animal models of lung injury are the administration of bleomycin (Moore and Hogaboam, 2008), acid (Modelska et al., 1999), or lipopolysaccharide (LPS) (Wiener-Kronish et al., 1991), all with characteristic advantages and disadvantages. This narrative review discusses molecular mechanisms in the development of experimental ARDS after LPS administration.

LPS is a part of the outer membrane of Gram-negative bacteria and can be administered into the airways (direct, pulmonary insult) and systemically (extra-pulmonary insult) (Menezes et al., 2005). The time-window used for induction and assessment of lung injury varies in the currently described models of LPS-induced lung injury. As lung injury evolves over time, molecular mechanisms of pathogenesis are differentially activated during disease progression. Molecular mechanisms that are essential during the early phase of LPS-induced lung injury may be less important for the late phase and vice versa. Translation of results from animal studies to more heterogeneous clinical studies failed for a lot of interventions in ARDS. This lack of clinical success could in part be attributed to differences in timing from injury to therapy onset in diverse patient populations. To define adequate timing of therapies in clinical studies, researchers must take the dynamics of respective successful animal model into account (Boyle et al., 2013).

## Lung Injury Induced by Lipopolysaccharide Challenge

LPS-induced lung injury is one of the most commonly used rodent models for ARDS (Matute-Bello et al., 2008) and was described to mimic the neutrophilic inflammatory response observed in ARDS patients (Matute-Bello et al., 2011).

Experimental evidence proposed that different pathophysiological pathways are activated during pulmonary and extra-pulmonary LPS challenge, especially during the early phase of disease progression (Menezes et al., 2005). Direct lung injury can be modeled in rodents by administration of LPS to the lungs through either tracheal instillation or inhalation. In this case, the alveolar epithelium is the primary structure that is damaged (Menezes et al., 2005). Local administration of LPS causes an acute and vigorous migration of inflammatory cells into the lung tissue with resolution by 72 h after the exposure (Bozinovski et al., 2004) followed by secondary fibrosis (Bitterman, 1992). Although this mechanism is not fully understood, LPS also affects alveolar type II cells and pulmonary surfactant through interaction with surfactant-specific proteins leading to their inactivation (Garcia-Verdugo et al., 2009; Glasser et al., 2009). Intravenous or intraperitoneal LPS administration triggers the release of inflammatory mediators into the systemic circulation, which in turn evokes indirect lung injury. In this case, the pulmonary vascular endothelium is the primary structure that is damaged and interstitial edema is the most prominent pathophysiological alteration. (Menezes et al., 2005). A single injection of LPS in the systemic environment is associated with relatively mild lung injury (Bastarache et al., 2009). Repeated or continuous LPS administration, however, has been shown to deteriorate lung injury in models of extra-pulmonary ARDS (Everhart et al., 2006; Cheng et al., 2007). Organ injury was also observed after intravenous injection of LPS (Wang et al., 2012) and is the most important cause of death in patients with ARDS according to clinical studies (Bersten et al., 2002; Estenssoro et al., 2002). So ARDS may be the cause or the consequence of organ failure suggesting a possible need for differential therapeutic approaches for ARDS management (Sorbo and Slutsky, 2011).

Pathophysiological, histological, and morphological differences found in pulmonary and extra-pulmonary ARDS may influence the response to pharmacological agents, mechanical ventilation, and positioning. Some studies have reported a modulation of the inflammatory process in ARDS in response to pharmacological therapies such as dasatinib (Oliveira et al., 2015) and corticosteroids (Leite-Junior et al., 2008) independent of the etiology. Clinical studies have evaluated ARDS treatments according to the underlying etiology. They found significant oxygenation improvement in pulmonary ARDS after inhaled nitric oxide (Rialp et al., 2001) and in extra-pulmonary ARDS after inhaled prostacyclin treatment (Domenighetti et al., 2001). Prone position improved oxygenation independent of the cause of ARDS in some studies (Rialp et al., 2001), while others showed benefits from positioning especially in patients with extra-pulmonary ARDS (Lim et al., 2001). These conflicting clinical data reflect different features of clinical ARDS: (1) different pathological factors in primary pulmonary and extra-pulmonary insult, affecting elastance and resistance of the lung and intra-abdominal pressure resulting in different responses to mechanical ventilation strategies; (2) different severity of injury; (3) the stage of ARDS (early or late); and (4) the difficulty to separate pulmonary and extra-pulmonary ARDS, which many times coexist.

## Molecular Mechanism of Lipopolysaccharide-Induced Lung Injury

**Figure 1:**
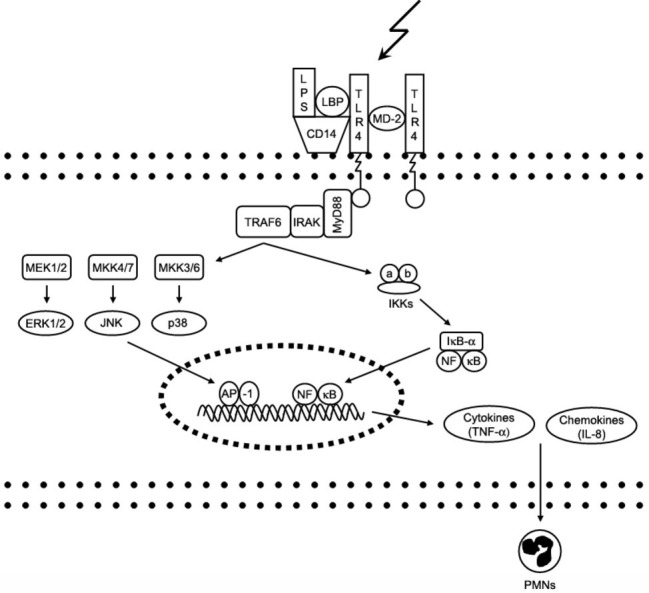
Signaling pathways of toll-like receptor 4. Interaction of lipopolysaccharide (LPS) with its receptor, toll-like receptor 4 (TLR-4), elicits strong innate immune responses through various intracellular signaling molecules. AP, activator protein; CD, cluster of differentiation; ERK, extracellular signal-regulated kinase; IkB, inhibitory kB; IKK, IkB kinase; IL, interleukin; IRAK, IL-1 receptor-associated kinase; JNK, c-Jun N-terminal kinase; LBP, LPS-binding protein; MD, myeloid differentiation protein; MEK, MKK, mitogen-activated protein kinase (MAPK) kinase; MyD, myeloid differentiation factor; NFkB, nuclear factor kB; PMNs, polymmorphonuclear leukocytes; TNF, tumor necrosis factor; TRAF6, TNF receptor-associated factor.

The cellular response to LPS is initiated by binding of LPS to the extracellular binding proteins LBP (LPS binding protein), CD14 and MD-2; this facilitates the binding of LPS to its main receptor – toll-like receptor 4 (TLR-4). Intracellular adaptor proteins (e.g., MyD88) bind to the TLR-4 receptor and activate various intracellular signaling cascades involving kinases like ERK1/2 (Extracellular signal-Regulated Kinase) and p38, which regulate the expression of cytokines with pro-inflammatory properties like TNF-α (tumor necrosis factor alpha) by activation of the NFkB (nuclear factor kappa-light-chain-enhancer of activated B cells) pathway (Medzhitov, 2007). The complex process of TLR-4 signaling has been reviewed in depth previously (Lu et al., 2008). Figure 1 shows major steps of LPS-triggered TLR-4 signaling.

## Differential Regulation of Lipopolysaccharide-Related Signaling Molecules Over Time

**Figure 2:**
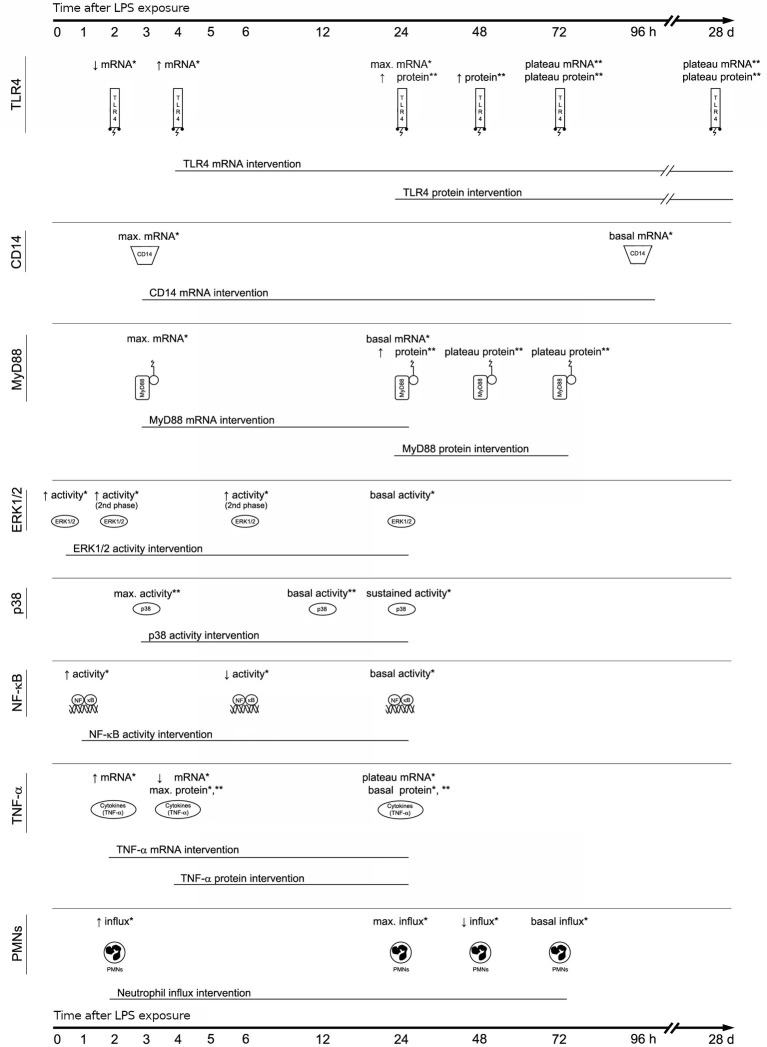
Lipopolysaccharide signaling pathways over time. Lipopolysaccharide (LPS) challenge affects pulmonary expression of signaling molecules over time (^*^pulmonary challenge or ^**^extra-pulmonary challenge). Not all signaling molecules are being activated at the same time, and not all signaling pathways are being activated for the same duration. So, rather confined therapeutic time-windows exist for effective targeting of LPS-induced signaling molecules. Changes in mRNA expression and protein concentration shown in hours/days post LPS challenge. CD, cluster of differentiation; ERK, extracellular signal-regulated kinase; mRNA, messenger ribonucleic acid; MyD, myeloid differentiation factor; NFkB, nuclear factor kB; PMNs, polymmorphonuclear leukocytes; TLR, toll-like receptor; TNF, tumor necrosis factor.

Experimental studies revealed that LPS challenge affects pulmonary expression of signaling molecules over time (Figure 2). For TLR-4, which acts as the main extracellular LPS receptor, kinetic studies demonstrated that mRNA expression initially decreased within 2 h after transnasal LPS challenge, increased at 4 h, and reached a maximum at 24 h (Bozinovski et al., 2004). At the protein level, TLR-4 was higher at 24, 48, and 72 h after intraperitoneal LPS challenge (Gao et al., 2012). TLR-4 mRNA and protein levels remained consistently elevated at 3 and 28 days after intraperitoneal LPS challenge (He et al., 2009). In contrast, molecules involved in the complex recognition of LPS by TLR-4 like CD14 and MD-2 showed a different pattern of activation. CD14 gene expression in the lung is rapidly increased to maximal levels at 3 h after intranasal LPS challenge, followed by a decrease to basal levels at 4 days, whereas MD-2 gene expression did not change after LPS exposure (Oshikawa and Sugiyama, 2003).

There is also evidence of differential activation of intracellular signal transduction pathways after LPS challenge. A kinetic study in mice described that pulmonary mRNA expression of the TLR-4 adaptor protein MyD88 was rapidly increased to maximal levels at 3 h after intranasal LPS challenge, followed by a decline to basal levels at 24 h (He et al., 2009). At the protein level, MyD88 expression was enhanced in the lung at 24 h after intraperitoneal LPS challenge (He et al., 2009). MyD88 protein levels remained nearly constant (i.e., plateau phase) over 48 and 72 h (He et al., 2009). Downstream, rapid activation of pulmonary ERK1/2 from the MAPK family was observed within 15 min after transnasal LPS challenge (Bozinovski et al., 2002). A minor secondary phase of ERK1/2 activation was observed between 2 and 6 h after LPS challenge decreasing to baseline levels at the end of the 24-h time course (Bozinovski et al., 2002). Considering the rapid response of ERK1/2 to LPS, ERK1/2 was proposed as a functional mediator in the TLR-4 signaling cascade (Bozinovski et al., 2002). In addition to ERK1/2, p38 MAPK has been recognized as an important mediator of LPS-induced lung injury. Phosphorylation of p38 MAPK rapidly increased, reached peak levels at 3 h, and slowly decreased to basal levels at 12 h after intravenous LPS challenge (Liu et al., 2008). In a model of intratracheal LPS however, phospho-p38 MAPK levels remained elevated at 24 h (Kim et al., 2006) supporting the hypothesis that pathophysiological pathways can be differentially activated during pulmonary or extra-pulmonary LPS challenge (Menezes et al., 2005).

Further downstream in the LPS-TLR-4 signaling cascade, transnasal LPS challenge was shown to affect DNA binding activity of NFkB over time. Pulmonary NFkB binding activity increased rapidly over the first hour after LPS challenge and slowly declined over 6 h, being resolved at the end of a 24-h period (Bozinovski et al., 2002). Accordingly, transnasal LPS challenge affected pulmonary TNF gene expression over time. After a steep increase within 2 h, TNF-α mRNA expression declined at 4 h after LPS challenge, without reaching baseline levels (Bozinovski et al., 2004). TNF-α mRNA expression remained nearly at a constant level for the rest of the 24-h period (Bozinovski et al., 2004). At the protein level, TNF-α expression peaked within 4 h after LPS challenge and slowly declined over 24 h (Bozinovski et al., 2004). A comparable 24-h TNF-α expression pattern was reported in mice exposed to intravenous LPS (Li et al., 2008). Finally, neutrophil infiltration into the lungs was evident within 2 h after transnasal LPS challenge in mice (Bozinovski et al., 2004). Neutrophil granulocytes increased over time, peaking at 24 h after LPS. The neutrophil levels markedly declined at 48 h and were resolved by 72 h (Bozinovski et al., 2004).

## Dynamics of Lipopolysaccharide-Induced Lung Injury

It has been shown that LPS elicits strong innate immune responses by activating various intracellular signaling pathways. In this regard, it is important that not all signaling molecules are being activated at the same time and not all signaling pathways are being activated for the same duration (Figure 2). For instance, TLR-4 protein expression was increased at 24 h after intraperitoneal LPS challenge (Gao et al., 2012) and remained consistently elevated at 3 and 28 days (He et al., 2009). Similarly, MyD88 protein levels were elevated at 24 h after intraperitoneal LPS challenge although remaining at a plateau level over 2–3 days (Oshikawa and Sugiyama, 2003). In complete contrast, MAPK and TNF-α showed a more transient expression pattern after intravenous LPS reaching peak levels within the first few hours and returning to basal levels within 12–24 h (Li et al., 2008; Liu et al., 2008). These experimental findings indicate that the pathophysiological pathways of LPS are time-dependent and imply that signaling molecules are differentially activated during disease progression. Besides the differential effect of time, different pathophysiological pathways are proposed to be activated during pulmonary or extra-pulmonary LPS challenge, at least during the early phase of disease progression (Menezes et al., 2005). Supporting this implication, prior kinetic studies showed that phosphorylation of p38 MAPK decreased to basal levels at 12 h after intravenous LPS challenge (extra-pulmonary insult) (Liu et al., 2008), whereas phospho-p38 MAPK levels remained elevated at 24 h after intratracheal LPS challenge (pulmonary insult) (Kim et al., 2006). These dynamics in LPS-induced lung injury are of utmost importance, particularly when developing or evaluating therapeutic strategies that target specific signaling molecules. Indeed, progression of lung injury was prevented when the neutrophil elastase inhibitor sivelestat was intravenously infused from 2 to 24 h after endotoxin inhalation (Kawabata et al., 2000), which is precisely the time-window of enhanced neutrophil infiltration after LPS challenge (Bozinovski et al., 2004). Concerning clinical cornerstones of ARDS therapy like positioning and fluid management, little is known on the effect of these interventions on experimental LPS-induced lung injury and its molecular dynamics.

In spite of the insights gained from the kinetic studies discussed here, only specific, limited time points after LPS challenge were examined. It may be possible that the duration of molecular responses is underestimated or a secondary phase of activation remains unnoticed. Most likely, different signaling molecules will be present in lung tissue at different time points after LPS challenge. Consequently, findings in rodent models of LPS-induced lung injury may depend on the time point at which samples are obtained and corresponding physiological data are captured.

## *In Vitro* Models of Lipopolysaccharide-Induced Lung Injury

The innate immune response of the lung to LPS is regulated by a complex association of receptors depending on cell type. *In vitro* studies using human alveolar epithelial cell line A549, and the human tracheobronchial epithelial cell lines BEAS-2B, showed the presence of TLRs at mRNA level, specifically TLR1–6 but not directly the TLR-4 (Schulz et al., 2002). The differences in TLR expression can depend on the stimulus, cell type, or subcellular location of the TLRs (McGettrick and O’Neill, 2010). It is already known that TLR-4 cycles between Golgi and plasma membrane (McGettrick and O’Neill, 2010) and that LPS response is regulated by the level of TLR-4 present on the cell surface membrane (Latz et al., 2002). Guillot et al. provided evidence for an intracellular compartmentalization of TLR-4 that allows LPS to induce the secretion of pro-inflammatory mediators in pulmonary epithelial cells. In this context, TLR-4 may be activated only by exposition to a high amount of LPS (Guillot et al., 2004).

Moreover, the presence of LPS does not appear to modulate the expression of TLR-4 in BEAS-2B cells incubated for short (1–6 h) or long period (48 h), yet is able to activate a TLR-4-dependent signaling pathway in this pulmonary epithelial cell (Guillot et al., 2004). As previously described, studies suggest the need of a coreceptors such as MD-2 and CD14 to initiate the binding between LPS and TLRs. *In vitro* studies showed that A549 and BEAS-2B express an LPS receptor that includes MD-2 but not CD14 (Guillot et al., 2004). Similar results were also observed in A549 but a with low expression of CD14 with BEAS-2B cells (Schulz et al., 2002), which can be explained by distinct basal activation or differentiation state of these cells or by a presence of a different mechanism involving CD14 on these cells in response to LPS. Despite these inconsistencies, LPS clearly induces the secretion of IL-8 and IL-6 in both cell lines in a concentration-dependent manner by involving the signal-transducing molecules MyD88, IRAK, TRAF6, and MAPK that activate the p38, Jnk, and ERK1/2 pathways (Guillot et al., 2004).

## Influence of Supportive Care on Lung Injury Dynamics

Lungs of ARDS patients are not only affected by the primary disease (e.g., pulmonary or extra-pulmonary insults) but also by therapeutic modalities used for supportive care (e.g., mechanical ventilation or fluid resuscitation). Accordingly, mechanical ventilation can be considered as a “second hit” in ARDS patients (Bregeon et al., 2002). This clinical condition can be mimicked in rodents by a “two-hit” model of lung injury combining mechanical ventilation with either pulmonary or extra-pulmonary LPS challenge. Previous experimental studies showed that mechanical ventilation interacts with endotoxemia, deteriorating lung injury (Altemeier et al., 2005; Bregeon et al., 2005) and promoting non-pulmonary organ failure (O’Mahony et al., 2006). Exposure to mechanical ventilation after LPS challenge therefore adds to the dynamics of LPS-induced lung injury. In this regard, it has been described that TLR-4 protein expression was already increased after intratracheal LPS challenge or mechanical ventilation alone, but was further enhanced when mechanical ventilation followed LPS challenge (Hu et al., 2010). Also downstream signaling molecules like cytokines and chemokines were shown to be higher when combining mechanical ventilation with LPS challenge (Haitsma et al., 2000; O’Mahony et al., 2006). It is worth noting that present technology complicates the use of prolonged supportive care in small animals. Therefore, available rodent models of mechanical ventilation may not mimic the clinical situation accurately where patients are ventilated for days or weeks.

## Perspective

Despite advances in understanding of dynamics of LPS-induced lung injury, additional *in vitro* studies should be performed to evaluate novel LPS-binding proteins in alveolar epithelial cells and further elucidate distinct pathways in response to LPS challenge. While the early response to LPS challenge is rather well characterized, there are few data on the late phase of LPS response.

This review describes the molecular mechanisms that contribute to lung injury in rodent models of LPS-induced organ injury mimicking either pulmonary or extra**-**pulmonary ARDS. Pathways of LPS-induced lung injury are not only affected by the type of the primary insult and the presence of additional stimuli but also by time. All these contributing factors should be taken into account when choosing a rodent model for developing and/or evaluating interventions that target specific pathophysiological pathways of LPS-induced lung injury. In this way, the therapeutic efficacy may be optimized and the translation of promising treatment strategies into patient care may be improved.

## Author Contributions

HD wrote the initial draft of the manuscript. MH composed the figures. All authors reviewed the available literature and summarized the data and reviewed and revised the final draft of the manuscript.

### Conflict of Interest

The authors declare that the research was conducted in the absence of any commercial or financial relationships that could be construed as a potential conflict of interest.
